# A Hyper-Pseudoelastic Model of Cyclic Stress-Softening Effect for Rubber Composites

**DOI:** 10.3390/polym15143033

**Published:** 2023-07-13

**Authors:** Yifeng Dong, Yutong Fu, Chunwang He, Daining Fang

**Affiliations:** 1 Institute of Advanced Structure Technology, Beijing Institute of Technology, Beijing 100081, China; dongyf369@163.com (Y.D.); fanggroup@bit.edu.cn (D.F.); 2 Beijing Key Laboratory of Lightweight Multi-Functional Composite Materials and Structures, Beijing Institute of Technology, Beijing 100081, China; 3 Collage of Aerospace Engineering, Chongqing University, Chongqing 400044, China

**Keywords:** cyclic stress-softening effect, strain energy evolution function, hyper-pseudoelastic model

## Abstract

Rubber composites are hyperelastic materials with obvious stress-softening effects during the cyclic loading–unloading process. In previous studies, it is hard to obtain the stress responses of rubber composites at arbitrary loading–unloading orders directly. In this paper, a hyper-pseudoelastic model is developed to characterize the cyclic stress-softening effect of rubber composites with a fixed stretch amplitude at arbitrary loading–unloading order. The theoretical relationship between strain energy function and cyclic loading–unloading order is correlated by the hyper-pseudoelastic model directly. Initially, the basic laws of the cyclic stress-softening effect of rubber composites are revealed based on the cyclic loading–unloading experiments. Then, a theoretical relationship between the strain energy evolution function and loading–unloading order, as well as the pseudoelastic theory, is developed. Additionally, the basic constraints that the strain energy evolution function must satisfy in the presence or absence of residual deformation effect are derived. Finally, the calibration process of material parameters in the hyper-pseudoelastic model is also presented. The validity of the hyper-pseudoelastic model is demonstrated via the comparisons to experimental data of rubber composites with different filler contents. This paper presents a theoretical model for characterizing the stress-softening effect of rubber composites during the cyclic loading–unloading process. The proposed theoretical model can accurately predict the evolution of the mechanical behavior of rubber composites with the number of loading–unloading cycles, which provides scientific guidance for predicting the durability properties and analyzing the fatigue performance of rubber composites.

## 1. Introduction

According to the participation of filler particles during the vulcanization process, rubber composites can be divided into the filled rubber composite and the unfilled rubber composite. The durability, toughness, and wear resistance of polymer composites can be significantly improved by incorporating filler particles [1,2,3] so that the filled rubber composites have been widely used in automobile tires, mechanical equipment, housing shock absorption structures, and aircraft door sealing [4,5]. Different from the unfilled rubber composites, the stress-softening effect and residual deformation are significant for the filled rubber composites, leading to the strong inelastic properties of filled rubber composites during the cyclic loading–unloading process [6,7]. Judging from the current research results, the magnitude of stress softening increases with the increase of the initial volume fraction of the filler particles [6,8]. In addition, the inelastic properties of the rubber composites vary greatly under different loading–unloading orders due to the residual deformation and the changes in internal structures [9]. Mullins [10,11] carried out systematic theoretical research and experimental tests on the stress-softening effect of rubber-like materials in the last century, so this phenomenon was named the Mullins effect. This phenomenon not only exists in the uniaxial tension [6,12,13,14] but also in the compression [15,16] and pure shear [17] and manifests as deformation-induced damage anisotropy [18]. Similar softening effects can also occur in other soft materials, such as hydrogels [19,20]. Because the stress-softening effect of rubber composites has an important influence on their fatigue damage characteristics and safe utilization, it is necessary to describe the basic physical characteristics of the stress-softening effect of rubber composites under cyclic loading–unloading processes. There are two main research ideas to study the stress-softening effect. One is the micromechanics theory, and the other is the phenomenological theoretical model.

In micromechanical theory, it is believed that the stress softening of rubber composites is caused by the following mechanisms: the breakdown of bonds between chains and filler particles [21,22], the internal sliding of the macromolecular chains, and the sliding of connecting chains on reinforcing filler particles [23], the breakage and re-agglomeration of the filler clusters [24], the aggregate-polymer debonding and network rearrangement [7], the damage of macromolecular chain [25], the rearrangement after macromolecular chain breakage [26], and chain delamination [27]. Govindjee et al. [21] introduced the concept of strain-induced debonding between the matrix and filler particles in the free energy density, assuming that the free energy of the polymer network could be decomposed into the free energies of chains in pure rubber network and chains distributed between filler particles. Additionally, the damage evolution process of the rubber composite is related to the maximum strain in the entire time history. In other words, if the current strain exceeds the maximum strain in the entire time history, further damage will occur. Subsequently, Horgan et al. [9] and Beatty et al. [28] reported that the stress-softening material has a memory effect on maximum deformation history. Göktepe et al. [22] and Miehe et al. [29] regarded the microstructure of rubber composites as being constructed by crosslink-to-crosslink (CC) networks and particle-to-particle (PP) networks. The stress-softening effect is mainly caused by a breakdown of bonds between chains and filler particles, and an anisotropic constitutive model for the description of the Mullins effect is proposed based on the non-affine micro-sphere model of rubber elasticity. Dargazany et al. [7] claimed that the inelastic behavior of the CC networks was negligible compared to the PP network, and the changes in the network structure included the aggregate-polymer debonding and network rearrangement during the loading process of rubber composites. Thus, a micromechanical network evolution theory was proposed to describe the damage mechanism of the internal network during the loading–unloading process based on the energy dissipation of the debonding chain and the orientation change of the chain distribution between the aggregates. According to the physical explanation of network rearrangement, Ayoub et al. [30] proposed a new network alteration theory to establish the relationship among the number of chain segments, chain density, and damage variables so as to characterize the stress-softening effect of rubber composite under large deformation. Zhu et al. [26] proposed the evolution laws of the network structure parameters for rubber composites by combining the micro-sphere model with the network alteration theory [12]. They analyzed the physical mechanism of the stress-softening effect of the rubber composites. Zhong et al. [25] proposed a quantitative model to characterize the anisotropic stress-softening effect of soft materials caused by the damage inhomogeneity during deformation. Although the micromechanical theory provides a reasonable physical explanation for the stress-softening effect, it is still difficult to describe the stress-softening effect of rubber composites accurately due to the complexity of the mechanical behaviors, such as the large deformation, nonlinear behavior, and viscoelasticity, etc.

The phenomenological theoretical model provides an appropriate way to investigate the stress-softening effect, even if there is no reasonable physical explanation for the phenomenological parameters. The phenomenological theoretical model does not specifically focus on the changes in the internal network structure of rubber composites but introduces some internal variable parameters, such as the damage variable, to describe the damage to the internal chain and microstructure and microcavity formation of rubber composites. In the phenomenological theoretical model proposed by Mullins et al. [1,31], the rubber composite was assumed to have a soft phase and a hard phase. Most of the strain occurs in the soft phase during deformation, but the proportion of the hard phase decreases continuously and transforms into the soft phase when the current stress exceeds the previous maximum stress. The stress-softening effect could be interpreted as the external manifestation of the internal damage of the rubber composite during the cyclic loading–unloading process. To this end, Lu et al. [32] introduced a rheological model considering the damage effects to characterize the stress-softening effect of soft materials. As for damage evolution, Simo [33] used the equivalent strain defined by the previous maximum strain energy to describe the damage evolution of rubber composites. In the last two decades, the most widely used phenomenological theoretical model is the pseudo-elasticity theory proposed by Dorfmann et al. [6]. In this theory, two internal variables are introduced to characterize the stress-softening effect and the residual deformation effect of rubber composites, respectively. Due to its excellent agreement with experimental results, the pseudo-elasticity theory has been embedded into the commercial finite element software Abaqus [34]. Based on the pseudo-elasticity theory, Dorfmann et al. [35] established the evolution law of the dissipation function and relaxation variables in the loading–unloading process and further deduced the stress response during loading, partial unloading, and reloading of the rubber composites. However, this model is only suitable for the case of no residual deformation, and the complexity of the dissipation function increases with the increase of loading–unloading order. Fazekas et al. [36,37] pointed out that the difference between loading response and unloading response in the presence of residual deformation was mainly caused by the Mullins effect and viscoelastic effect, so a hyper-visco-pseudoelastic model was established by combining the pseudoelastic theory with the viscoelastic theory.

In conclusion, it can be found that the existing theoretical models are mainly used to characterize the stress-softening effect of the single loading–unloading process. However, there are few studies focusing on the stress-softening effect during cyclic loading–unloading processes with a fixed stretch amplitude, and the mechanical response changes caused by the cyclic loading are not taken into account. In this paper, the concept of strain energy evolution function is proposed, and a hyper-pseudoelastic model with cyclic loading–unloading order *N* is established. Thus, the nominal stress–stretch curve corresponding to different loading–unloading orders is characterized quantitatively. The research results have important guiding significance for the durability of rubber composites. The rest of the paper is organized as follows: in Section 2, the basic laws of stress-softening effect are revealed based on the cyclic loading–unloading experiments. Then, the phenomenological hyper-pseudoelastic model is established in Section 3. Furthermore, the application of the hyper-pseudoelastic model, including the model validation and parameter calibration, is presented in Section 4. Finally, several important conclusions are drawn in Section 5.

## 2. Basic Laws of Stress-Softening Effect

**Figure 1:**
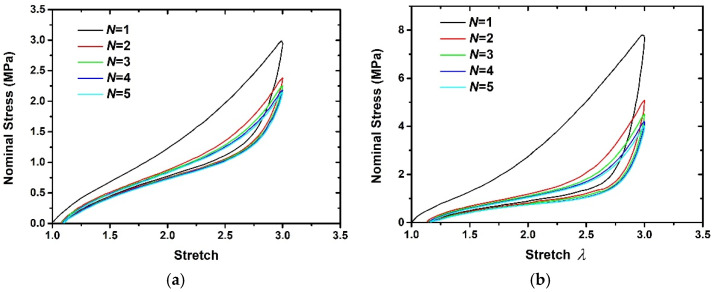
The nominal stress–stretch curves under different loading–unloading order [6]. (**a**) 20 phr filled rubber composite, (**b**) 60 phr filled rubber composite.

The nominal stress–stretch curves of 1 phr (by volume), 20 phr, and 60 phr filled rubber composites are tested by Dorfmann et al. [6] during loading–unloading cyclic at the temperature of 25 °C, the strain rate of 0.02 s^−1^.The cross-sectional dimension of the specimen is 2 × 4 mm in the initial state. The specimen was subjected to cyclic loading–unloading with constant strain amplitude and the maximum principal stretch λ=3. Since the nominal stress–stretch curves after the fifth loading–unloading cycle are essentially repeatable with negligible additional stress softening and residual deformation, only the nominal stress–stretch curves for the first five loading–unloading cycles are plotted here. Compared with the 20 phr and 60 phr filled rubber composites, the stress-softening effect of 1 phr filled rubber composite is negligible. In order to demonstrate the basic laws of the stress-softening effect under different loading–unloading orders, the experimental results corresponding to the 20 phr and 60 phr filled rubber composites are taken here, as shown in Figure 1. From the detailed observation of Figure 1, some basic laws are summarized as follows:

(1) The area enclosed by the loading–unloading curve gradually decreases with the increase of the cyclic loading–unloading order.
(2) In the presence of residual deformation, a negative load must be applied to completely restore the rubber composites to a non-deformed state; that is, the unloading nominal stress is less than 0 when the principal stretch λ equals 1. In addition, the residual deformation increases with the increase of the loading–unloading order, and the residual deformation of the rubber composites mainly occurs during the first loading–unloading process.
(3) The nominal stress difference corresponding to the previous loading–unloading curve is greater than that corresponding to the subsequent loading–unloading curve under the same stretch, which actually confirms the basic law (1).
(4) The previous loading curves are always above the subsequent loading curves, and the unloading curves also have the same feature. And there is an intersection point between the previous unloading curve and the subsequent loading curve.
(5) When the maximum stretch amplitude remains unchanged, the stress-softening effect corresponding to the first loading–unloading process is the most obvious. After several loading–unloading, the stress response tends to be stable. A similar phenomenon was also observed by Dorfmann et al. [18], Simo [33], and Sasso et al. [38].
(6) Compared with 20 phr filled rubber composite, 60 phr filled rubber composite has a more obvious stress-softening effect, which indicates that the stress-softening effect is more significant with the increase of filler content in rubber composites.

## 3. Hyper-Pseudoelastic Model of Cyclic Stress-Softening Effect

**Figure 2:**
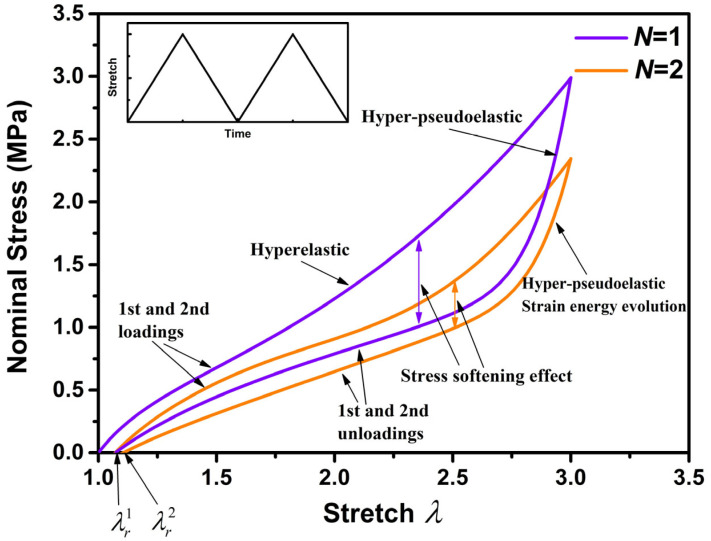
The stress-softening effect in cyclic loading–unloading of rubber composite.

The nominal stress–stretch curve of the rubber composite for the first two cycles is presented, as shown in Figure 2. It can be observed from Figure 2 that the rubber composite cannot recover to its initial state after unloading. In addition, both the first and second cycles exhibit stress-softening effects, but the theoretical models used to describe the stress-softening effects in these two cycles differ. The stress-softening effect during the first loading–unloading is mainly attributed to pseudoelasticity, while the stress-softening effect during the second loading–unloading is influenced not only by pseudoelasticity but also by the evolution of strain energy. It is necessary to establish a new hyper-pseudoelastic model to investigate the stress-softening effect of rubber composite during cyclic loading–unloading.

Dorfmann–Ogden model [6] introduced two internal variables $\eta_{1},\eta_{2}$ into the hyperelastic strain energy model to characterize the stress-softening effect and residual deformation during loading–unloading process, which could characterize the stress-softening effect of rubber composites during single loading–unloading process well. But this model could not characterize the cyclic loading–unloading characteristics of rubber composites. A large number of experimental results show that there are differences in the mechanical properties of rubber composites not only in the loading process and unloading process but also in different loading–unloading orders. For this purpose, the strain energy evolution function $\varphi (N,\lambda_{1},\lambda_{2},\lambda_{3})$ considering the loading–unloading order *N* is introduced into Dorfmann–Ogden model to investigate the cyclic stress-softening effect of rubber composites. The rubber composites are regarded as incompressible and isotropic, and the specific form of strain energy function $W_{N}(N,\lambda_{1},\lambda_{2},\lambda_{3},\eta_{1},\eta_{2})$ in the cyclic loading–unloading process is given as follows:

(1) $$\begin{array}{l} W_{N}(N,\lambda_{1},\lambda_{2},\lambda_{3},\eta_{1},\eta_{2})=W(\lambda_{1},\lambda_{2},\lambda_{3},\eta_{1},\eta_{2})\varphi (N,\lambda_{1},\lambda_{2},\lambda_{3})\\ \;\;=\left[\eta_{1}W_{0}(\lambda_{1},\lambda_{2},\lambda_{3})+(1-\eta_{2})R(\lambda_{1},\lambda_{2},\lambda_{3})+\phi_{1}(\eta_{1})+\phi_{2}(\eta_{2})\right]\varphi (N,\lambda_{1},\lambda_{2},\lambda_{3}) \end{array}$$

where $\left(\lambda_{1},\lambda_{2},\lambda_{3}\right)$ are the principal stretches of rubber composite. For incompressible rubber composite, $\lambda_{1}\lambda_{2}\lambda_{3}=1$. $W_{0}(\lambda_{1},\lambda_{2},\lambda_{3})$ is the original strain energy function of rubber composite. $\eta_{1}=\eta_{2}=1$ in the loading process and $\eta_{1},\eta_{2}$ depend on $\left(\lambda_{1},\lambda_{2},\lambda_{3}\right)$ during the unloading process. $R(\lambda_{1},\lambda_{2},\lambda_{3})$ is used to characterize the residual deformation effect and $\phi_{1}(\eta_{1}),\phi_{2}(\eta_{2})$ denote the dissipation function. *N* is the loading–unloading order. The value range of strain energy evolution function is $0<\varphi (N,\lambda_{1},\lambda_{2},\lambda_{3})\leq 1$. In a sense, the strain energy evolution function is equal to (1-*D*), and *D* represents the damage variable in the continuum of damage mechanics.

According to the explanation proposed by Ogden et al. [39,40], the non-recoverable dissipation energy is equal to the area enclosed by the loading curve and unloading curve, which may be interpreted as a measure of the energy required to cause the damage in the rubber composite. Based on the principle of energy conservation, the non-recoverable dissipation energy of rubber composite during the previous *N*-1 loading–unloading process can be expressed as the following form:

(2) $$W_{diss}(N-1)=H(N)\sum_{i=1}^{N-1}W(\lambda_{1r}^{i},\lambda_{2r}^{i},\lambda_{3r}^{i},\eta_{1r}^{i},\eta_{2r}^{i})\varphi (i,\lambda_{1r}^{i},\lambda_{2r}^{i},\lambda_{3r}^{i})$$

where $\left(\lambda_{1r}^{i},\lambda_{2r}^{i},\lambda_{3r}^{i}\right)$ represent the residual principal stretch, and $\eta_{1r}^{i},\eta_{2r}^{i}$ are the corresponding internal variables during the *i*-th unloading process. H(N) has the following form:

(3) $$H(N)=\left\{ \begin{array}{l} 1,\;N>1\\ 0,\;N=1 \end{array}\right.$$

For the uniaxial loading state, $\lambda_{2}=\lambda_{3}=\lambda_{1}^{-\frac{1}{2}}$ can be carried out by the incompressible characteristics of rubber composite, Equations (1) and (2) can be expressed as follows:

(4) $$\begin{array}{l} W_{N}(N,\lambda ,\eta_{1},\eta_{2})=W(\lambda ,\eta_{1},\eta_{2})\varphi (N,\lambda )\\ \;\;\;\;\;\;\;=\left[\eta_{1}W_{0}(\lambda )+(1-\eta_{2})R(\lambda )+\phi_{1}(\eta_{1})+\phi_{2}(\eta_{2})\right]\varphi (N,\lambda )\\ W_{diss}(N-1)=H(N)\sum_{i=1}^{N-1}W(\lambda_{r}^{i},\eta_{1r}^{i},\eta_{2r}^{i})\varphi (i,\lambda_{r}^{i}) \end{array}$$

### 3.1. No Residual Deformation Effect

It is assumed here that the rubber composites do not have any residual deformation after complete unloading. Although there is usually more or less residual deformation after unloading for most rubber composites, the stress-softening effect of rubber composites can be better evaluated without considering the residual deformation effect, and it can provide a basis for the investigation of the residual deformation effect. According to the pseudo-elasticity theory [6,12,13,14], $\eta_{1}$ is a monotonically increasing function of λ, and $\eta_{1r}^{i}=\eta_{1\min}^{i}$ when the principal stretch $\lambda_{r}^{i}=1$ after the *i*-th unloading. Based on Equation (4), the strain energy function and the dissipation energy under uniaxial cyclic loading–unloading without residual deformation can be obtained as follows:

(5) $$\begin{array}{l} W_{N}(N,\lambda ,\eta_{1})=\left[\eta_{1}W_{0}(\lambda )+\phi_{1}(\eta_{1})\right]\varphi (N,\lambda )\\ W_{diss}(N-1,1,\eta_{1\min}^{i})=H(N)\sum_{i=1}^{N-1}\left[\eta_{1\min}^{i}W_{0}(1)+\phi_{1}(\eta_{1\min}^{i})\right]\varphi (i,1) \end{array}$$

Because $\eta_{1}=1$ and $\phi_{1}(1)=0$ in the loading process, the nominal stress $t_{L}^{N}$ can be obtained in the following form:

(6) $$t_{L}^{N}=\frac{\partial W_{N}(N,\lambda ,1)}{\partial \lambda }=t_{0}\varphi (N,\lambda )+W_{0}(\lambda )\frac{\partial \varphi (N,\lambda )}{\partial \lambda }$$

The nominal stress $t_{U}^{N}$ in the unloading process has the following form:

(7) $$t_{U}^{N}=\frac{\partial W_{N}(N,\lambda ,\eta_{1})}{\partial \lambda }=\eta_{1}t_{0}\varphi (N,\lambda )+\left[\eta_{1}W_{0}(\lambda )+\phi_{1}(\eta_{1})\right]\frac{\partial \varphi (N,\lambda )}{\partial \lambda }$$

Since the nominal stress is 0 after complete unloading (λ=1), the nominal stress in the whole loading–unloading process must satisfy $t_{L}^{N}\geq t_{U}^{N}\geq 0$. According to Equations (6) and (7), $t_{L}^{N}$ and $t_{U}^{N}$ must satisfy the following:

(8) $$\begin{array}{l} t_{L}^{N}-t_{U}^{N}=(1-\eta_{1})t_{0}\varphi (N,\lambda )+\left[W_{0}(\lambda )-(\eta_{1}W_{0}(\lambda )+\phi_{1}(\eta_{1}))\right]\frac{\partial \varphi (N,\lambda )}{\partial \lambda }\geq 0\\ t_{U}^{N}=\eta_{1}t_{0}\varphi (N,\lambda )+\left[\eta_{1}W_{0}(\lambda )+\phi_{1}(\eta_{1})\right]\frac{\partial \varphi (N,\lambda )}{\partial \lambda }\geq 0 \end{array}$$

According to the pseudo-elasticity theory [6,12,13,14], $\eta_{1}$ and $\phi_{1}(\eta_{1})$ have the following forms, respectively:

(9) $$\eta_{1}=1-\frac{1}{r}\tanh\left[\frac{W_{m}-W_{0}(\lambda )}{\mu m}\right]$$

(10) $$\phi_{1}(\eta_{1})=-\mu m(\eta_{1}-1)\tanh^{-1}\left[r(\eta_{1}-1)\right]-W_{m}(\eta_{1}-1)-\frac{\mu m}{2r}\ln\left[1-r^{2}{\left(\eta_{1}-1\right)}^{2}\right]$$

Here, r and m are dimensionless positive pseudoelastic material parameters and μ denotes the shear modulus of the rubber composite in initial configuration. $W_{m}=W_{0}(\lambda_{m})$ represents the maximum strain energy during the loading process, where $\lambda_{m}$ denotes the maximum principal stretch.

Because 0<φ(N,λ)≤1, and

(11) $$W_{0}(\lambda )-(\eta_{1}W_{0}(\lambda )+\phi_{1}(\eta_{1}))=\frac{\mu m}{2r}\ln\left[1-r^{2}{\left(\eta_{1}-1\right)}^{2}\right]<0$$

According to Equation (8), φ(N,λ) must satisfy

(12) $$\frac{-\eta_{1}t_{0}}{\eta_{1}W_{0}(\lambda )+\phi_{1}(\eta_{1})}\leq \frac{\partial \varphi (N,\lambda )}{\partial \lambda }/\varphi (N,\lambda )\leq \frac{-(1-\eta_{1})t_{0}}{W_{0}(\lambda )-\left[\eta_{1}W_{0}(\lambda )+\phi_{1}(\eta_{1})\right]}$$

Since $\frac{\partial \varphi (N,\lambda )}{\partial \lambda }/\varphi (N,\lambda )$ is either a constant or a function of *N*. And

(13) $${\left[\frac{-\eta_{1}t_{0}\varphi (N,\lambda )}{\eta_{1}W_{0}(\lambda )+\phi_{1}(\eta_{1})}\right]}_{\max}={\left[\frac{-(1-\eta_{1})t_{0}\varphi (N,\lambda )}{\left[W_{0}(\lambda )-(\eta_{1}W_{0}(\lambda )+\phi_{1}(\eta_{1}))\right]}\right]}_{\min}=0$$

Therefore, in the case of no residual deformation, in order for Equation (12) to be satisfied under arbitrary loading–unloading order and arbitrary principal stretch, the following constraints must be satisfied:

(14) $$\frac{\partial \varphi (N,\lambda )}{\partial \lambda }=0$$

Thus, the following formula can be obtained:

(15) φ(N,λ)=φ(N)

According to Equation (5)_2_, the dissipation energy in the first loading–unloading process is:

(16) $$W_{diss}^{1}=W_{N}(1,1,\eta_{1\min}^{1})=\left[\eta_{1\min}W_{0}(1)+\phi_{1}(\eta_{1\min}^{1})\right]\varphi (1)=\phi_{1}(\eta_{1\min}^{1})$$

Since $\eta_{1}$ is positively related to the principal stretch λ, $\eta_{1\min}^{1}$ is the internal variable value at λ=1. Similarly, the dissipation energy $W_{diss}^{N}$ generated during the *N*-th loading–unloading process is

(17) $$W_{diss}^{N}=W_{N}(N,1,\eta_{1\min}^{N})=\phi_{1}(\eta_{1\min}^{N})\varphi (N)$$

According to the basic law (1), there is the following relationship:

(18) $$\phi_{1}(\eta_{1\min}^{N})\varphi (N)>\phi_{1}(\eta_{1\min}^{N+1})\varphi (N+1)$$

Since there is no residual deformation after the rubber composite is completely unloaded, $\eta_{1\min}^{N}=\eta_{1\min}^{N+1}$, $\phi_{1}(\eta_{1\min}^{N})=\phi_{1}(\eta_{1\min}^{N+1})$. Then, the following property of strain energy evolution function can be obtained:

(19) φ(N)>φ(N+1)

As shown in the above equation, φ(N) a monotonically decreasing function with the loading–unloading order *N*.

Basic law (3) summarizes the relationship of nominal stress during the cyclic loading–unloading process. According to Equations (6) and (7), it can be known:

(20) $$\begin{array}{l} \Delta t^{N}=t_{L}^{N}-t_{U}^{N}=(1-\eta_{1})t_{0}\varphi (N)\\ \Delta t^{N+1}=t_{L}^{N+1}-t_{U}^{N+1}=(1-\eta_{1})t_{0}\varphi (N+1) \end{array}$$

According to Equation (19), $\Delta t^{N}\geq \Delta t^{N+1}$ is always satisfied under any principal stretch. It can be found that as long as the basic law (3) is satisfied, the basic law (1) can be deduced. Similarly, $t_{L}^{N}>t_{L}^{N+1},t_{U}^{N}>t_{U}^{N+1}$ can be further deduced.

To satisfy the above requirements, an exponential expression is chosen to represent φ(N):

(21) $$\varphi (N)=1-\sum_{i=1}^{n}g_{i}(1-e^{\frac{1-N}{A_{i}}})$$

where $g_{i}$ and $A_{i}$ are dimensionless positive material parameters. The value range of $g_{i}$ is (0,1].

It can be seen from basic law (4) that there is an intersection point between the subsequent loading curve and the previous unloading curve, that is, when $\lambda =\lambda_{e}^{N}$, $t_{U}^{N}=t_{L}^{N+1}$. $\lambda_{e}^{N}$ can be obtained by solving the following equation:

(22) $$\eta_{1}(\lambda_{e}^{N})\varphi (N)=\varphi (N+1)$$

### 3.2. Residual Deformation Effect

As described in basic law (2), in order to fully restore the rubber composite to a non-deformation state, a negative load must be applied in the presence of residual deformation. Here, $\eta_{2}$ and $\phi_{2}(\eta_{2})$ are introduced into the strain energy function to describe the residual deformation effect. Because of the existence of residual deformation, the initial stretch of the subsequent loading is the residual stretch of the previous unloading, which will affect the mechanical characteristics of the subsequent loading–unloading. Therefore, it is necessary to consider the influence of the previous residual stretch on the strain energy function of the subsequent loading–unloading process. Thus, the strain energy function and dissipation energy $W_{diss}$ during the cyclic loading–unloading process are given as follows:

(23) $$\begin{array}{l} W_{N}(N,\lambda ,\eta_{1}^{N},\eta_{2}^{N})=\left[\eta_{1}^{N}W_{0}(\lambda -\lambda_{r}^{N-1}+1)+(1-\eta_{2}^{N})R(\lambda -\lambda_{r}^{N-1}+1)+\phi_{1}(\eta_{1}^{N})+\phi_{2}(\eta_{2}^{N})\right]\varphi (N,\lambda -\lambda_{r}^{N-1}+1)\\ W_{diss}(N-1)=H(N)\sum_{i=1}^{N-1}\left[\eta_{1r}^{i}W_{0}(\lambda_{r}^{i}-\lambda_{r}^{i-1}+1)+(1-\eta_{2r}^{i})R(\lambda_{r}^{i}-\lambda_{r}^{i-1}+1)+\phi_{1}(\eta_{1r}^{i})+\phi_{2}(\eta_{2r}^{i})\right]\varphi (i,\lambda_{r}^{i}-\lambda_{r}^{i-1}+1) \end{array}$$

Here, $\lambda_{r}^{i}$ represents the residual principal stretch of *i*-th unloading. Here, the initial state is still considered the reference state. Since the subsequent loading process is performed on the basis of $\lambda_{r}^{i}$, the relative principal stretch $\lambda_{R}^{N}$ of the subsequent loading–unloading process is given by

(24) $$\lambda_{R}^{N}=\lambda -\lambda_{r}^{N-1}+1$$

which means that the principal stretch of the subsequent loading–unloading process needs to consider the residual principal stretch after the previous unloading. Because the $\lambda_{R}^{N}$ is related to the previous loading–unloading processes, a Matlab code is written to calculate $\lambda_{R}^{N}$ for arbitrary loading–unloading process. The residual principal stretch $\lambda_{r}^{N-1}$ is reflected through the relative principal stretch $\lambda_{R}^{N}$ in $\eta_{1}^{N}$ and $\eta_{2}^{N}$. Thus, $\eta_{1}^{N}$ and $\phi_{1}(\eta_{1}^{N})$ specialize to

(25) $$\eta_{1}^{N}=1-\frac{1}{r}\tanh\left[\frac{W_{m}^{N}-W_{0}(\lambda_{R}^{N})}{\mu m}\right]$$

(26) $$\phi_{1}(\eta_{1}^{N})=-\mu m(\eta_{1}^{N}-1)\tanh^{-1}\left[r(\eta_{1}^{N}-1)\right]-W_{m}^{N}(\eta_{1}^{N}-1)-\frac{\mu m}{2r}\ln\left[1-r^{2}{\left(\eta_{1}^{N}-1\right)}^{2}\right]$$

where $W_{m}^{N}=W_{0}(\lambda_{Rm}^{N})$. $\lambda_{Rm}^{N}=\lambda_{m}-\lambda_{r}^{N-1}+1$ represents the maximum relative principal stretch.

As $W_{m}^{N}>W_{m}^{N+1}$, $\lambda_{r}^{N}>\lambda_{r}^{N-1}$, the residual principal stretch increases with the increase of loading–unloading order.

According to the pseudo-elasticity theory [6,12,13,14], the specific forms of $\eta_{2}$ and $\phi_{2}(\eta_{2})$ are as follows:

(27) $$\eta_{2}^{N}=\tan h\left[{\left(\frac{W_{0}(\lambda_{R}^{N})}{W_{m}^{N}}\right)}^{a+b*W_{m}^{N}/\mu }\right]/\tanh(1)$$

(28) $$\phi_{2}{}^{\prime }(\eta_{2}^{N})=R(\lambda_{R}^{N})$$

where *a* and *b* are the material parameters.

Since it is difficult to obtain an explicit expression for $\phi_{2}(\eta_{2}^{N})$, we use numerical integration to compute $\phi_{2}(\eta_{2}^{N})$ with $\phi_{2}(1)=0$.

Due to $\eta_{1}=\eta_{2}=1$ and $\phi_{1}(1)=\phi_{2}(1)=0$ in the loading process, the corresponding nominal stress $t_{L}^{N}$ has the following form:

(29) $$t_{L}^{N}=\frac{\partial W_{N}(N,\lambda ,1,1)}{\partial \lambda }=t_{0}(\lambda_{R}^{N})\varphi (N,\lambda_{R}^{N})+W_{0}(\lambda_{R}^{N})\frac{\partial \varphi (N,\lambda_{R}^{N})}{\partial \lambda }$$

The nominal stress $t_{U}^{N}$ in the unloading process has the following form:

(30) $$\begin{array}{l} t_{U}^{N}=\frac{\partial W_{N}(N,\lambda ,\eta_{1}^{N},\eta_{2}^{N})}{\partial \lambda }\\ \;\;\;\;\;=\left[\eta_{1}^{N}t_{0}(\lambda_{R}^{N})+(1-\eta_{2}^{N})R^{\prime }(\lambda_{R}^{N})\right]\varphi (N,\lambda_{R}^{N})+\left[\eta_{1}^{N}W_{0}(\lambda_{R}^{N})+(1-\eta_{2}^{N})R(\lambda_{R}^{N})+\phi_{1}(\eta_{1}^{N})+\phi_{2}(\eta_{2}^{N})\right]\frac{\partial \varphi (N,\lambda_{R}^{N})}{\partial \lambda } \end{array}$$

Because $t_{L}^{N}\geq t_{U}^{N}$, the following constraint must be satisfied:

(31) $$\begin{array}{l} \left[t_{0}(\lambda_{R}^{N})-\left[\eta_{1}^{N}t_{0}(\lambda_{R}^{N})+(1-\eta_{2}^{N})R^{\prime }(\lambda_{R}^{N})\right]\right]\varphi (N,\lambda_{R}^{N})\geq \\ -\left[W_{0}(\lambda_{R}^{N})-\left[\eta_{1}^{N}W_{0}(\lambda_{R}^{N})+(1-\eta_{2}^{N})R(\lambda_{R}^{N})+\phi_{1}(\eta_{1}^{N})+\phi_{2}(\eta_{2}^{N})\right]\right]\frac{\partial \varphi (N,\lambda_{R}^{N})}{\partial \lambda } \end{array}$$

In the above formula,

(32) $$W_{0}(\lambda_{R}^{N})-\left[\eta_{1}^{N}W_{0}(\lambda_{R}^{N})+(1-\eta_{2}^{N})R(\lambda_{R}^{N})+\phi_{1}(\eta_{1}^{N})+\phi_{2}(\eta_{2}^{N})\right]\leq 0$$

Here, the equal sign is taken when $\lambda_{R}^{N}=\lambda_{Rm}^{N}$. When $\lambda_{r}^{N-1}\leq \lambda <\lambda_{m}$, the following inequalities need to be satisfied:

(33) $$\frac{\partial \varphi (N,\lambda_{R}^{N})}{\partial \lambda }/\varphi (N,\lambda_{R}^{N})<-\frac{t_{0}(\lambda_{R}^{N})-\left[\eta_{1}^{N}t_{0}(\lambda_{R}^{N})+(1-\eta_{2}^{N})R^{\prime }(\lambda_{R}^{N})\right]}{W_{0}(\lambda_{R}^{N})-\left[\eta_{1}^{N}W_{0}(\lambda_{R}^{N})+(1-\eta_{2}^{N})R(\lambda_{R}^{N})+\phi_{1}(\eta_{1}^{N})+\phi_{2}(\eta_{2}^{N})\right]}$$

In addition, $t_{L}^{N}\geq 0$ when $\lambda_{r}^{N-1}\leq \lambda <\lambda_{m}$, the equal sign is taken when $\lambda =\lambda_{r}^{N-1}$, so that

(34) $$\frac{\partial \varphi (N,\lambda_{R}^{N})}{\partial \lambda }/\varphi (N,\lambda_{R}^{N})\geq -\frac{t_{0}(\lambda_{R}^{N})}{W_{0}(\lambda_{R}^{N})}$$

Combined with Equations (33) and (34), the constraints of the $\varphi (N,\lambda_{R}^{N})$ are

(35) $$-\frac{t_{0}(\lambda_{R}^{N})}{W_{0}(\lambda_{R}^{N})}\leq \frac{\partial \varphi (N,\lambda_{R}^{N})}{\partial \lambda }/\varphi (N,\lambda_{R}^{N})<-\frac{t_{0}(\lambda_{R}^{N})-\left[\eta_{1}^{N}t_{0}(\lambda_{R}^{N})+(1-\eta_{2}^{N})R^{\prime }(\lambda_{R}^{N})\right]}{W_{0}(\lambda_{R}^{N})-\left[\eta_{1}^{N}W_{0}(\lambda_{R}^{N})+(1-\eta_{2}^{N})R(\lambda_{R}^{N})+\phi_{1}(\eta_{1}^{N})+\phi_{2}(\eta_{2}^{N})\right]}$$

## 4. Results and Discussion

The Ogden model [41,42] is used to characterize the hyperelastic properties of rubber composites, and the specific form is as follows:

(36) $$W_{0}(\lambda_{1},\lambda_{2},\lambda_{3})=\sum_{j=1}^{J}\frac{\mu_{j}}{\alpha_{j}}(\lambda_{1}^{\alpha_{j}}+\lambda_{2}^{\alpha_{j}}+\lambda_{3}^{\alpha_{j}}-3)$$

where $\mu_{j},\alpha_{j}$ are the phenomenological hyperelastic material parameters, which is obtained by fitting the first loading curve. For uniaxial loading, $\lambda_{2}=\lambda_{3}=\lambda_{1}^{-\frac{1}{2}}$, the above strain energy function becomes

(37) $$W_{0}(\lambda )=\sum_{j=1}^{J}\frac{\mu_{j}}{\alpha_{j}}(\lambda^{\alpha_{j}}+2\lambda^{-\alpha_{j}/2}-3)$$

According to the above formula, the nominal stress under uniaxial loading can be obtained:

(38) $$t_{0}=\sum_{j=1}^{J}\mu_{j}(\lambda^{\alpha_{j}-1}-\lambda^{-\alpha_{j}/2-1})$$

where $\mu_{j},\alpha_{j}$ can be determined by the initial loading curve. The hyperelastic material parameters $\mu_{j},\alpha_{j}$ corresponding to 20 phr and 60 phr filled rubber composites are listed in Table 1, where *J* = 3. The initial shear modulus $\mu =0.5\sum_{j=1}^{J}\mu_{j}\alpha_{j}$.

Dorfmann et al. [6] suggested the modified neo-Hookean model to represent the specific expression of $R(\lambda_{1},\lambda_{2},\lambda_{3})$:

(39) $$R(\lambda_{1},\lambda_{2},\lambda_{3})=0.5\left[\nu_{1}(\lambda_{1}^{2}-1)+\nu_{2}(\lambda_{2}^{2}-1)+\nu_{3}(\lambda_{3}^{2}-1)\right]$$

For uniaxial loading, the above expression becomes

(40) $$R(\lambda )=0.5\left[\nu_{1}(\lambda^{2}-1)+2{\bar{\nu }}_{2}(\lambda^{-1}-1)\right]$$

where ${\bar{\nu }}_{2}=(\nu_{2}+\nu_{3})/2$.

$\nu_{1},{\bar{\nu }}_{2}$, r,m, and a,b are the pseudoelastic material parameters, which can be determined by the initial unloading curve. The fitting process for hyperelastic and pseudoelastic material parameters is as follows: first, Equation (38) is used to fit the first loading curve to calibrate the hyperelastic material parameters. Then, with the hyperelastic material parameters fixed, Equation (30) is used to fit the first unloading curve to calibrate the pseudoelastic material parameters. Finally, based on the fitting results of the two steps mentioned above, the initial values of the hyperelastic and pseudoelastic material parameters are set, and these material parameters are fine-tuned. The hyperelastic and pseudoelastic material parameters of 20 phr and 60 phr filled rubber composites are listed in Table 1.

Combined with Equations (36)–(40), the cyclic loading–unloading characteristics of rubber composites in the presence or absence of residual deformation effect can be carried out according to the hyper-pseudoelastic model.

### 4.1. No Residual Deformation Effect

According to the theoretical model presented in Section 3.1, Equation (21) is used to describe the cyclic loading–unloading characteristics of rubber composites without residual deformation. Here, *n* = 1 to simplify the complexity of parameter analysis, Equation (21) becomes the following form:

(41) $$\varphi (N)=1-g(1-e^{\frac{1-N}{A}})$$

**Figure 3:**
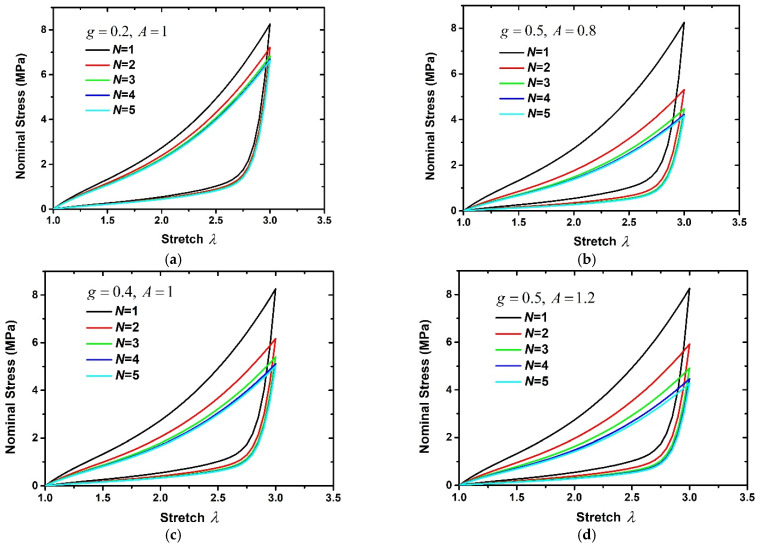

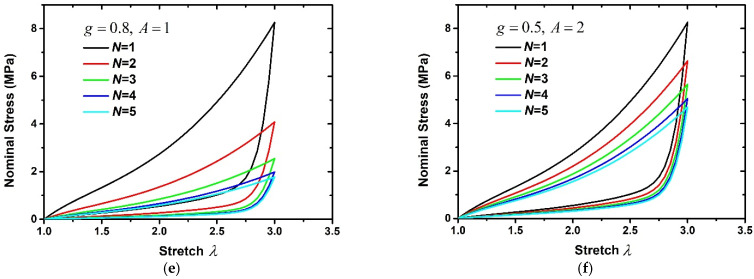
The stress–stretch curves corresponding to different *A* and *g.* (**a**) g=0.2, A=1, (**b**) g=0.5, A=0.8, (**c**) g=0.4, A=1, (**d**) g=0.5, A=1.2, (**e**) g=0.8, A=1, (**f**) g=0.5, A=2.

**Figure 4:**
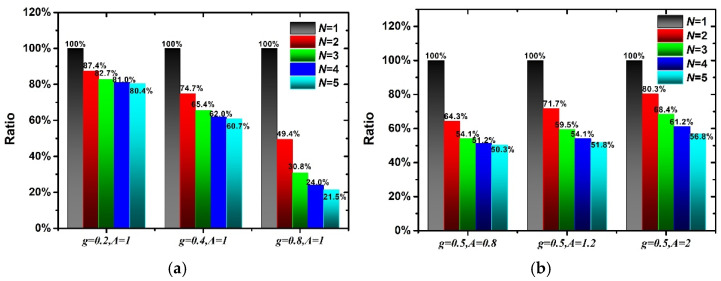
The area changes of loading–unloading curves corresponding to different *A* and *g*. (**a**) Different *g*, (**b**) Different *A*.

Here, the influences of *A* and *g* on the stress-softening effect are discussed. The hyperelastic and pseudoelastic material parameters of 60 phr filled rubber composite are used in this section. Figure 3 shows the nominal stress–stretch curves corresponding to different *A* and *g*. It can be found from Figure 3a,c,e that the larger *g* means that more cyclic loading–unloading times are needed to reach a stable stress state and a larger reduction in the maximum stress. When *g* is fixed, the larger *A* means that more cyclic loading–unloading times are also needed to reach a stable stress state. But the stress–stretch curves shown in Figure 3b,d,f are only slightly different. Therefore, *g* mainly controls the stress-softening effect of rubber composites, while *A* mainly plays a role in fine-tuning the stress–stretch curve. In addition, the area enclosed by the loading curve and unloading curve represents the dissipation energy during the loading–unloading process. In order to compare the effects of *g* and *A* on the dissipation energy, Figure 4 illustrates the ratio of the dissipated energy in the subsequent loading cycle to that in the first loading cycle corresponding to different *g* and *A*. It can be found that the dissipation energy decreases the most, reaching 78.5% when *g* = 0.8, *A* = 1. And the dissipation energy is larger when *g* is smaller, or *A* is larger corresponding to the same loading–unloading order.

### 4.2. Residual Deformation Effect

Based on the theoretical model in Section 3.2 and the experimental results shown in Figure 1, this section will conduct the parameter calibration and the deduction of specific expression of strain energy function considering the residual deformation effect. According to the research results in Section 3.1, the strain energy evolution function considering the influence of residual deformation is given in the following form:

(42) $$\varphi (N,\lambda_{R}^{N})=\left[1-g(1-e^{\frac{1-N}{A}})\right]\times e^{\beta (\lambda_{R}^{N})}$$

In order to satisfy the constraints of Equation (35) and the value range of the strain energy evolution function, $\beta (\lambda_{R}^{N})$ must satisfy the following constraints:

(43) $${\left[-\frac{t_{0}(\lambda_{R}^{N})}{W_{0}(\lambda_{R}^{N})}\right]}_{\max}\leq \frac{d\beta (\lambda_{R}^{N})}{d\lambda_{R}^{N}}<{\left[-\frac{t_{0}(\lambda_{R}^{N})-\left[\eta_{1}^{N}t_{0}(\lambda_{R}^{N})+(1-\eta_{2}^{N})R^{\prime }(\lambda_{R}^{N})\right]}{W_{0}(\lambda_{R}^{N})-\left[\eta_{1}^{N}W_{0}(\lambda_{R}^{N})+(1-\eta_{2}^{N})R(\lambda_{R}^{N})+\phi_{1}(\eta_{1}^{N})+\phi_{2}(\eta_{2}^{N})\right]}\right]}_{\min}$$

(44) $$\beta (\lambda_{R}^{N})<-\ln\left[1-g(1-e^{-\frac{1}{A}})\right]$$

The specific expression form of $\beta (\lambda_{R}^{N})$ needs to be determined according to the nominal stress–stretch curve of the rubber composite. The specific expression form of $\beta (\lambda_{R}^{N})$ will be complicated when the nominal stress–stretch curve is complex or when the nonlinear properties of the loading nominal stress–stretch curve and the unloading nominal stress–stretch curve are quite different. Additionally, it can be seen from Equations (29) and (30) that the unloading stress formula naturally has stronger nonlinear characteristics than the loading stress formula. Therefore, a more complicated expression of $\beta (\lambda_{R}^{N})$ is required when describing loading nominal stress–stretch curve, while a more concise form can be chosen to describe unloading nominal stress–stretch curve. Here, $\beta (\lambda_{R}^{N})$ is given as the following form:

(45) $$\begin{array}{l} \beta (\lambda_{R}^{N})=\left[k_{1}+k_{2}(1+sign(\lambda_{R}^{\prime N}))(k_{3}{\left(\lambda_{Rm}^{N}-\lambda_{R}^{N}\right)}^{3}+k_{4}{\left(\lambda_{Rm}^{N}-\lambda_{R}^{N}\right)}^{2})\right]\times \\ \;\;\;\left[k_{5}{\left(\lambda_{R}^{N}\right)}^{4}+k_{6}{\left(\lambda_{R}^{N}\right)}^{3}+k_{7}{\left(\lambda_{R}^{N}\right)}^{2}+k_{8}\lambda_{R}^{N}+k_{9}\right] \end{array}$$

where $k_{i},i=1,2\ldots 9$ are the material parameters of strain energy evolution function, which are determined by fitting the cyclic loading–unloading experimental curve. *sign*() denotes the signum function, which is used to distinguish between the loading process and unloading process. $\lambda_{R}^{\prime N}=\frac{d\lambda_{R}^{N}}{dt}$ represents the derivative of the relative principal stretch with respect to time, which is positive during the loading process and negative during the unloading process. It is worth mentioning that its specific form of $\beta (\lambda_{R}^{N})$ is not fixed, and it can be chosen according to the mechanical behavior of rubber composites. The selection principle for $\beta (\lambda_{R}^{N})$ is mainly as follows: firstly, it should ensure that $\beta (\lambda_{R}^{N})$ can accurately describe the nonlinearity of the loading–unloading curve, while minimizing the complexity of the $\beta (\lambda_{R}^{N})$ and reducing the number of fitting parameters as much as possible. Secondly, it should ensure the continuity of both $\beta (\lambda_{R}^{N})$ and its derivative $\frac{d\beta (\lambda_{R}^{N})}{d\lambda_{R}^{N}}$ at the maximum stretch $\lambda_{Rm}^{N}$. Thirdly, $\beta (\lambda_{R}^{N})$ must satisfy Equations (43) and (44).

**Figure 5:**
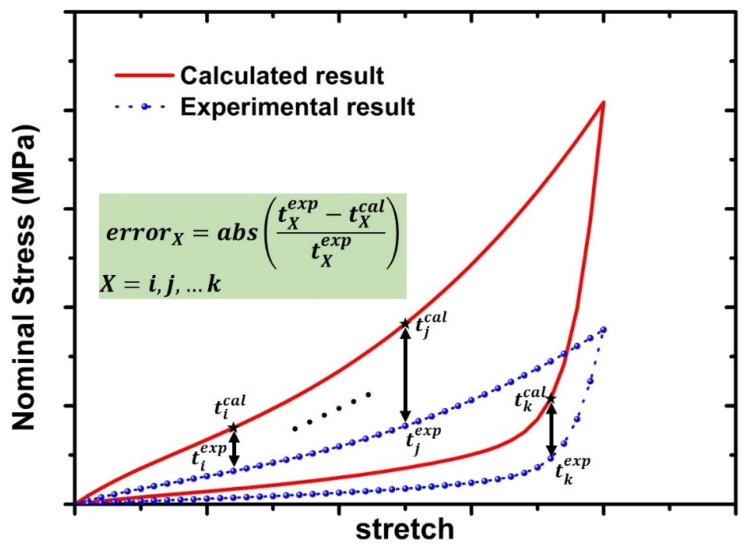
Schematic diagram of relative error calculation.

Figure 1 records the cyclic loading–unloading mechanical behavior of 20 phr and 60 phr filled rubber composites, which are used for parameters calibration and model verification. Here, the first three loading–unloading curves are used to determine the above material parameters, and the last two loading–unloading curves are used to verify the hyper-pseudoelastic model. The calibration process of material parameters is as follows: First, only the unloading nominal stress–stretch curve is fitted using Equation (30); the values of $k_{2},k_{3},k_{4}$ can be given arbitrarily and fixed so that the material parameters $k_{1},k_{5},k_{6},k_{7},k_{8},k_{9},g,A$ can be obtained. Then, fix the parameters $k_{1},k_{5},k_{6},k_{7},k_{8},k_{9},g,A$ obtained in the first step. The material parameters $k_{2},k_{3},k_{4}$ can be obtained by fitting the loading nominal stress–stretch curve using Equation (29). Finally, set the initial value of material parameters based on the fitting results of the above two steps, and fine-tune those material parameters. Figure 5 shows the calculation diagram of optimization objectives during the parameter fitting. Here, the calculated results of the cyclic loading–unloading curve are computed at each optimization and compared with the experimental results. The following formula is used to estimate the relative error between calculated results and experimental results and serves as the optimization objective value during the fitting process. The Matlab code is written to realize the above fitting process, in which the constraint conditions of Equations (43) and (44) are incorporated into the Matlab code to ensure that the $\beta (\lambda_{R}^{N})$ always satisfy Equations (43) and (44). The interior-point algorithm [43] is used to minimize the optimization objective value.

(46) $$Error=\sum_{i=1}^{K}\left(abs\left(\frac{t_{i}^{\exp}-t_{i}^{cal}}{t_{i}^{\exp}}\right)\right)$$

where *K* is the number of experimental data points and $t_{i}^{\exp}$, $t_{i}^{cal}$ are the experimental and calculated stress at the same stretch, respectively.

**Figure 6:**
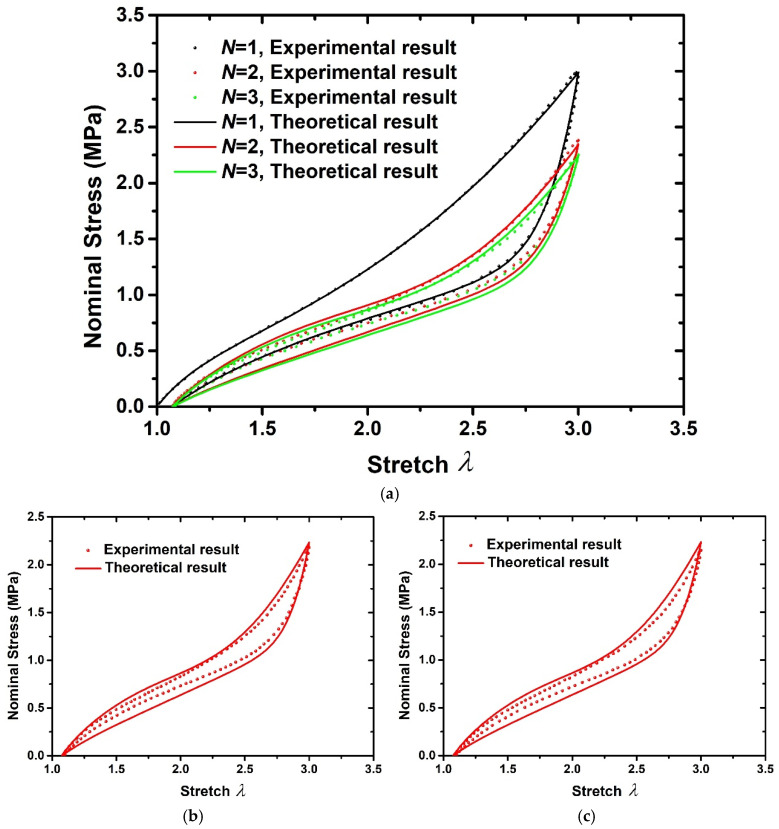
Comparison of experimental results [6] and theoretical results of 20 phr filled rubber composite. (**a**) *N* = 1,2,3, (**b**) *N* = 4, (**c**) *N* = 5.

**Figure 7:**
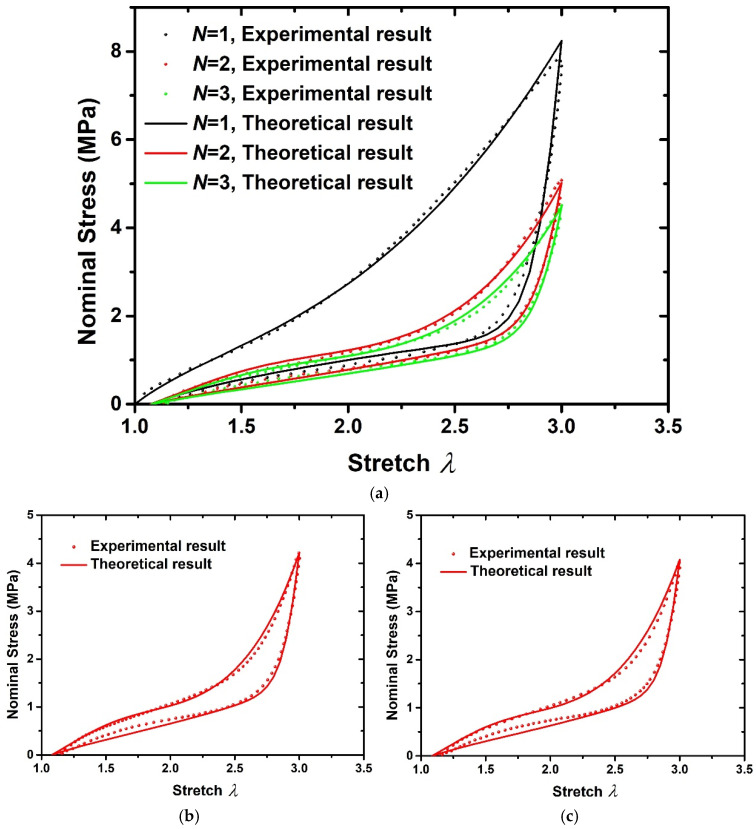
Comparison of experimental results [6] and theoretical results of 60 phr filled rubber composite. (**a**) *N* = 1,2,3, (**b**) *N* = 4, (**c**) *N* = 5.

Figure 6 and Figure 7 show the comparison of experimental and theoretical results of 20 phr and 60 phr filled rubber composites, respectively. Figure 6a and Figure 7a show the experimental results and fitting results of the first three loading–unloading curves of 20 phr and 60 phr filled rubber composites. Thus, the material parameters g,A and $k_{i},i=1,2\ldots 9$ can be determined, as shown in Table 2. The 4th and 5th loading–unloading curves are drawn using the above theoretical formula, and the comparison with the experimental results are drawn in Figure 6b,c and Figure 7b,c, respectively. It can be seen from Figure 6b,c and Figure 7b,c that the model is capable of predicting the stress softening behavior of subsequent loading–unloading cycles by means of the fitting parameters of the first three cycles, which proves that the hyper-pseudoelastic model can effectively characterize the cyclic loading–unloading characteristics of rubber composites with residual deformation, and has good applicability for rubber composites with different filler contents.

## 5. Conclusions

In this paper, a hyper-pseudoelastic model is developed to characterize the cyclic stress-softening effect of rubber composites based on the pseudo-elasticity theory. In addition, the cyclic stress-softening effects of rubber composites in the presence or absence of residual deformation effects are discussed. The specific conclusions are summarized as follows:

(1) A detailed analysis of the cyclic loading–unloading experimental results of rubber composites has been carried out. The basic laws of stress-softening effect are summarized to guide the derivation of the hyper-pseudoelastic model. It can be found that the stress-softening effect and residual deformation of rubber composites corresponding to different loading–unloading orders are different, especially the nominal stress–stretch curves of initial loading–unloading and subsequent loading–unloading are significantly different.
(2) The hyper-pseudoelastic model in the cyclic loading–unloading process is proposed, in which a strain energy evolution function similar to (1-*D*) in continuum damage mechanics is introduced to characterize the cyclic stress-softening effect. The specific expressions of the strain energy evolution function with or without residual deformation effect are given, and the constraints that the strain energy evolution function needs to satisfy are also obtained based on the basic laws of the stress-softening effect. The hyper-pseudoelastic model establishes the theoretical relationship between strain energy and cyclic loading–unloading order directly, which provides great convenience in deriving the stress response corresponding to arbitrary loading–unloading order.
(3) The influences of the material parameters on the cyclic loading–unloading curve are discussed. The research results show that *g* mainly controls the degree of stress softening of rubber composites, while *A* mainly plays a role in fine-tuning the stress–stretch curve. Additionally, the dissipation energy is larger when *g* is smaller, or *A* is larger corresponding to the same loading–unloading order.
(4) Based on the nominal stress–stretch experimental results of cyclic loading–unloading processes, the calibration method of material parameters and specific expression of strain energy evolution function with residual deformation effect are obtained. Further, the hyper-pseudoelastic model is verified by comparing the theoretical results with experimental results. The proposed model can predict the cyclic stress-softening effect of rubber composites with different filler contents effectively.

## Figures and Tables

**Table 1:** The material parameters of 20 phr and 60 phr filled rubber composites.

20 phr	Hyperelastic material parameters					
	$\mu_{1}(\mathrm{MPa})$	$\alpha_{1}$	$\mu_{2}(\mathrm{MPa})$	$\alpha_{2}$	$\mu_{3}(\mathrm{MPa})$	$\alpha_{3}$
	0.05	4.221	−0.815	−0.156	−0.286	−4.63
	Pseudoelastic material parameters					
	$\nu_{1}(\mathrm{MPa})$	${\bar{\nu }}_{2}(\mathrm{MPa})$	r	m	a	b
	0.491	0.596	0.963	0.753	0.438	1.952
60 phr	Hyperelastic material parameters					
	$\mu_{1}(\mathrm{MPa})$	$\alpha_{1}$	$\mu_{2}(\mathrm{MPa})$	$\alpha_{2}$	$\mu_{3}(\mathrm{MPa})$	$\alpha_{3}$
	−1.528	−1.011	0.223	4.205	−1.134 × 10^−3^	−4.399
	Pseudoelastic material parameters					
	$\nu_{1}(\mathrm{MPa})$	${\bar{\nu }}_{2}(\mathrm{MPa})$	r	m	a	b
	0.354	0.496	1.25	0.965	0.3	0.16

**Table 2:** The material parameters of strain energy evolution function for 20 phr and 60 phr filled rubber composites.

20 phr	g	A	$k_{1}$	$k_{2}$	$k_{3}$	$k_{4}$
	0.466	0.343	−0.011	0.028	−0.085	−0.136
	$k_{5}$	$k_{6}$	$k_{7}$	$k_{8}$	$k_{9}$	
	0.468	−0.526	−7.003	2.46	−0.375	
60 phr	g	A	$k_{1}$	$k_{2}$	$k_{3}$	$k_{4}$
	0.34	1.33	−0.08	0.104	−0.173	0.375
	$k_{5}$	$k_{6}$	$k_{7}$	$k_{8}$	$k_{9}$	
	0	0.479	−3.688	5.479	8.99	

## Data Availability

The data presented in this study are available on request from the corresponding author.
